# Inflaming the Clock: Effects of Interleukin 1β on Circadian Rhythmicity of Pancreatic β Cells

**DOI:** 10.1210/endocr/bqaa115

**Published:** 2020-07-07

**Authors:** Alexandre Martchenko, Patricia L Brubaker

**Affiliations:** 1 Department of Physiology, University of Toronto, Toronto, Ontario, Canada; 2 Department of Medicine, University of Toronto, Toronto, Ontario, Canada

**Keywords:** circadian, inflammation, insulin, interleukin 1β, secretion, type 2 diabetes

Circadian rhythms are endogenous 24-hour anticipatory rhythms that are present in all known organisms (1). These rhythms developed over evolutionary time to anticipate predefined environmental changes such as the light-dark cycle, and are regulated by a transcriptional:translational feedback loop that exists between the core positive (Bmal1/Clock) and negative (Period/Cryptochrome) proteins (1). In mammals, light is the strongest zeitgeber, or “time giver,” entraining the master clock in the suprachiasmatic nuclei of the hypothalamus which, in turn, send out time-dependent signals to synchronize peripheral tissues (1). Interestingly, metabolic tissues, including the pancreatic islets, have also been shown to express cell-autonomous rhythms, driven in part by other zeitgebers such as nutrient intake (2, 3). Importantly, circadian disruption through genetic or environmental factors has implicated the pancreatic β cell clock as an important mediator of metabolic homeostasis through regulation of circadian insulin secretion (4, 5). Human epidemiological data further suggest an important link between circadian rhythms and metabolism, as individuals who conduct shift-work are at increased risk of developing type 2 diabetes (T2D) (6). However, whether disruption of the β cell clock is causative to development of T2D or is merely a consequence of the disease remains to be determined. To this end, Javeed et al (7) have investigated the role of inflammatory cytokines that are characteristic to conditions of metabolic dysfunction, on the pancreatic β cell clock. Using a wide variety of models ranging from β cell lines to human islets, Javeed et al demonstrate disruptive effects of the pro-inflammatory cytokine interleukin-1β (IL-1β) on the β cell clock (7), thereby providing a potential link between disruption of the β cell clock and T2D.

Isolation of islets from transgenic mice expressing a mouse insulin promoter (MIP)-driven Period2-luciferase:green fluorescence reporter construct (*Per2*-LUC:GFP) allowed for tracking of circadian rhythmicity within β cells upon treatment with various pro-inflammatory cytokines. Interestingly, exposure to IL-1β, but not to other cytokines with known roles in causing β cell damage (tumor necrosis factor-α [TNFα], IL-6, and interferon-γ), resulted in significant dampening of the amplitude and shortening of the phase of the β cell circadian rhythm, as evidenced by *Per-*driven changes in bioluminescence. Functionally, islets exposed to IL-1β also lost their rhythmic pattern in glucose-stimulated insulin secretion (GSIS) in association with a reduced amplitude of release. However, it is important to note that although IL-1β disrupts the circadian architecture of the β cell, exposure to this diabetogenic cytokine also reduces β cell function overall, making it difficult to conclude that the effects of IL-1β are mediated exclusively through the clock. To strengthen the conclusion that IL-1β–induced β cell clock disruption results in the abrogated GSIS pattern, it may be important to study circadian GSIS upon treatment with other pro-inflammatory cytokines that do not have a disruptive effect on the β cell clock.

To examine the mechanism by which IL-1β disrupts circadian β cell function Javeed et al (7) measured expression of the genes for Bmal1 and Clock (*Arntl* and *Clock*) in the INS-1 β cell line. Interestingly, not only IL-1β but also TNFα and IL-6 impaired *Arntl* expression, with largely no effect on *Clock*. These data speak to the importance of using multiple models, given the lack of effects of TNFα and IL-6 on islets from the *Per2*:LUC-MIP:GFP mice. Further study established that IL-1β impairs *Arntl* promoter activity and BMAL1 protein expression. Furthermore, IL-1β was found to decrease the expression of sirtuin-1 (SIRT1), a positive regulator of *Arntl*, and this was reversed by reactivation of SIRT1 using resveratrol. Interestingly, although IL-1β disruption and SIRT1 reactivation did not affect GSIS in β cell Bmal1 knockout islets, IL-1β treatment increased basal (4 mM) but not stimulated (16 mM) insulin secretion, thereby dampening the glucose stimulation index. These data suggest that IL-1β may be responsible for preventing the fold increase in GSIS from basal rather than GSIS in general.

Finally, to extend their findings beyond IL-1β-mediated circadian β cell disruption, islets from both a diabetic mouse model and humans with T2D were studied (7). Mild streptozotocin-induced β cell damage was found to completely abrogate β cell circadian rhythmicity, as evidenced by a reduction in the amplitude of *Per2* expression. However, although streptozotocin does result in a pro-inflammatory state, this mouse model does not fully recapitulate the development of T2D, due to the absence of peripheral insulin resistance. However, evidence of decreased expression of BMAL1, as well as of several clock regulators, including sirtuin 1 (SIRT1), in β cells from humans with T2D confirmed findings of a recent study detailing circadian clock dysfunction in these cells (8). Hence, previous work (8) and the present study by Javeed et al (7) definitively establish an association between a disrupted β cell clock and T2D. However, although genetic models have shown that β cell clock disruption can cause diabetes in mice, one outstanding question in the field remains to be answered: which comes first in humans, impaired circadian gene expression or T2D?

In summary, the work by Javeed et al (7) provides extensive evidence for a novel role of inflammation and, specifically the cytokine IL-1β, as a stressor of the circadian architecture of the β cell. These findings add to the growing list of factors that induce circadian disruption of β cells. It will be important to elucidate if circadian disruption of cells and tissues that are the sources of inflammatory cytokines leads to a pro-inflammatory state, downstream of which is impaired function of metabolic tissues such as the pancreatic β cells. Finally, future studies will have to focus on modeling the progression of subjects with normoglycemia through impaired glucose tolerance and on to T2D to determine if circadian dysregulation of β cells is a major causative factor for T2D.

## Abbreviations

GSIS: glucose-stimulated insulin secretion
IL-1β: interleukin-1β
T2D: type 2 diabetes
TNFα: tumor necrosis factor-α

## Additional Information

***Disclosure Summary:*** The authors have no relevant conflicts of interest to disclose.

***Data Availability:*** Data sharing is not applicable to this article as no datasets were generated or analyzed during the current study.
